# Biosafety at Home: How to Translate Biomedical Laboratory Safety Precautions for Everyday Use in the Context of COVID-19

**DOI:** 10.4269/ajtmh.20-0677

**Published:** 2020-06-26

**Authors:** Miguel Reina Ortiz, Mario J. Grijalva, Michael J. Turell, William F. Waters, Andres Carrazco Montalvo, Derrick Mathias, Vinita Sharma, Christian Fierro Renoy, Paul Suits, Stephen J. Thomas, Renato Leon

**Affiliations:** 1 College of Public Health, University of South Florida, Tampa, Florida;; 2 Department of Biomedical Sciences, Infectious and Tropical Disease Institute, Heritage College of Osteopathic Medicine, Ohio University, Athens, Ohio;; 3 Center for Research on Health in Latin America (CISeAL), Facultad de Ciencias Exactas y Naturales, Pontificia Universidad Católica del Ecuador, Quito, Ecuador;; 4 VectorID LLC, Frederick, Maryland;; 5 School of Public Health, Universidad San Francisco de Quito, Quito, Ecuador;; 6 Medical Entomology and Tropical Medicine Laboratory LEMMT, School of Biological and Environmental Sciences, Universidad San Francisco de Quito, Quito, Ecuador;; 7 Florida Medical Entomology Laboratory, University of Florida, Vero Beach, Florida;; 8 College of Public Health and Health Professions, University of Florida, Gainesville, Florida;; 9 College of Medicine, University of Florida, Gainesville, Florida;; 10 Hospital Metropolitano de Quito, Quito, Ecuador;; 11 SUNY Upstate Medical University, Syracuse, New York

## Abstract

Population adoption of social distancing measures during the COVID-19 pandemic is at times deficient, increasing the risk of SARS-CoV-2 transmission. Healthcare workers and those living in areas of intense transmission may benefit from implementing biosafety measures in their daily lives. A mixed-methods approach, combining components of single negotiation text and the Delphi method, was used to create a COVID-19 biosafety-at-home protocol. A consensus building coordinator liaised with 12 experts to develop the protocol over 11 iterations. Experts had more than 200 years of combined experience in epidemiology, virology, infectious disease prevention, and public health. A flyer, created from the final protocol, was professionally designed and initially distributed via social media and institutional websites/emails in Ecuador beginning on May 2, 2020. Since then, it has been distributed in other countries, reaching ∼7,000 people. Translating research laboratory biosafety measures for the home/street environment might be challenging. The biosafety-at-home flyer addresses this challenge in a user-friendly format.

As of June 16, 2020, more than 7.5 million cases and more than 400,000 deaths due to COVID-19 have been reported worldwide.^1^ Coronavirus pandemic epicenters have shifted from Asia to Europe and the Americas.^1^ In Latin America, Ecuador ranks sixth in absolute number of COVID-19 cases^1^ and fourth in per capita rates (281 per 100,000 population) using population data obtained from the World Bank.^2^ These figures, however, are largely believed to be an underestimation of the real spread of SARS-CoV-2,^3^ the causative agent of COVID-19. Furthermore, Ecuador has started phasing out containment measures, which may lead to subsequent epidemic peaks.^4^

Social distancing measures, effective to stop SARS-CoV-2 transmission,^5^ have not always been thoroughly followed. Recent reports suggest a loose adoption of social distancing measures in Guayaquil,^6,7^ the largest and most affected city of Ecuador. The combination of large community spread^1^ with improper adoption of social distancing^6^ has been of concern to the population. Indeed, mental health issues may arise in the context of the COVID-19 pandemic and its related lockdowns.^8^ In addition, social media has been inundated with inaccurate advice.^9^ In this scenario, there is need for professionals to provide scientifically proven information that can minimize the risk of contagion and people’s anxiety, particularly among high-risk groups such as healthcare workers (HCWs)^10^ or those living in areas of intense transmission.

In response to this need, a group of scientists with broad experience in public health, laboratory studies, and field research on infectious diseases (IDs) developed a protocol that translates research laboratory biosafety precautions to everyday life. The protocol is intended for use among high-risk groups such as those living in areas of intense transmission and HCWs,^10,11^ especially those working in hospital areas where SARS-CoV-2 is likely to be transmitted.^12^

A user-friendly flyer derived from this protocol was made available via social media. Here, we report on protocol development as well as on initial flyer distribution. We also provide an electronic copy of the protocol in English (Supplemental Materials, Protocol S1) and of the flyer in Spanish (Supplemental Materials, Flyer S1).

Although developed because of pressing needs in Ecuador, the biosafety-at-home flyer was created with the idea that it could potentially be useful in other countries. For instance, Brazil has surpassed the United Kingdom, Spain, Italy, and Russia in terms of COVID-19 cases.^1^ Recently, an initiative to translate the biosafety-at-home protocol to Portuguese has been launched.

We used a mixed approach based on single negotiation text (SNT) and the Delphi method (DM) to develop a consensus on how to translate biosafety-level (BSL) measures to the home/street environment to reduce exposure to SARS-CoV-2. The SNT process includes proposing a written statement, soliciting repeated input, and ultimately agreeing on a final statement.^13^ The DM consists of developing consensus from expert opinion based on a similar process that is anonymous, iterative, and provides controlled feedback and summary statistics—4-point Likert scales have effectively been used in the DM.^14^ Although an anonymous and iterative process with controlled feedback was followed, we did not include a Likert scale or any other similar survey format to collect experts’ input.

Our mixed-methods approach amalgamated SNT and DM processes into a consensus development model structured, as shown in Figure 1. A consensus building coordinator (CBC) was in charge of 1) selecting experts; 2) receiving, compiling, and incorporating feedback into the developing consensus document; and 3) liaising with professional graphic designers for final product development.

**Figure 1.**
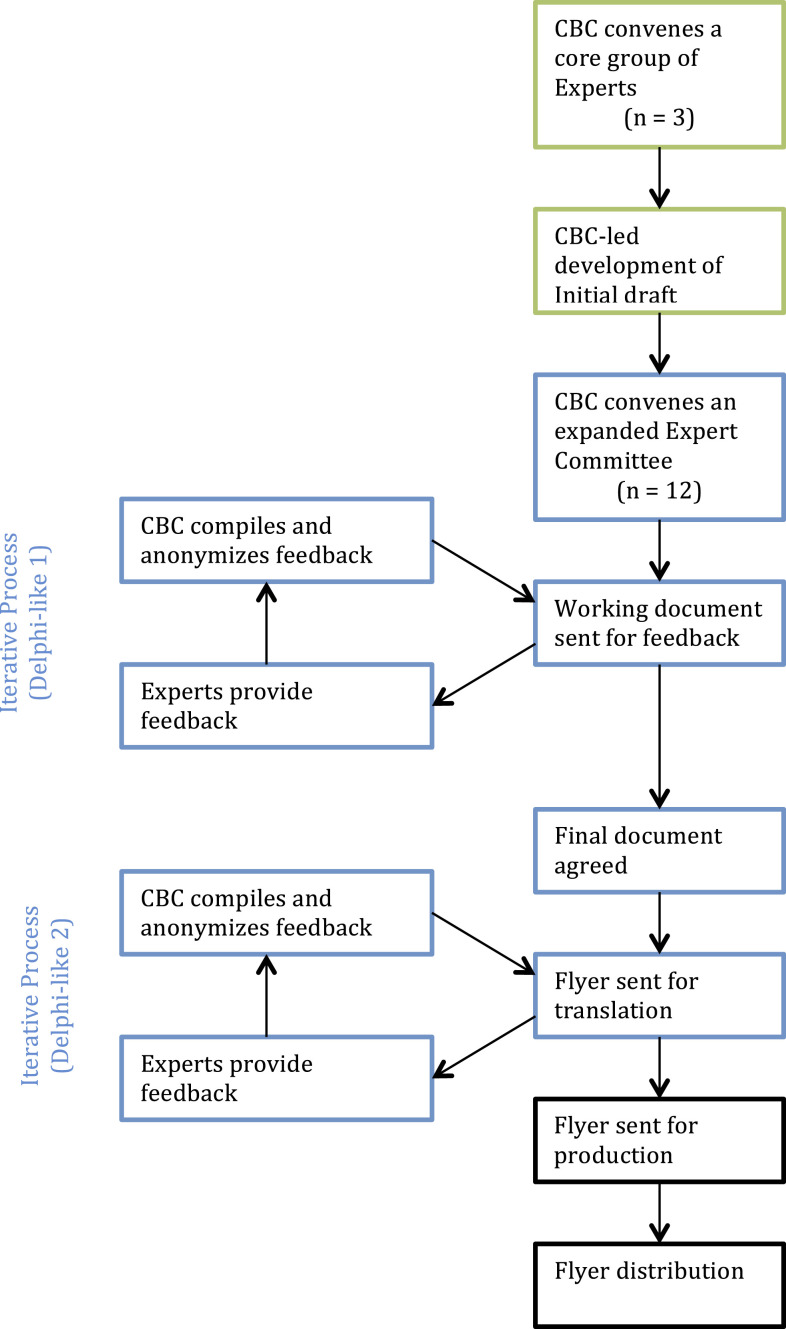
Development of biosafety-at-home flyer. Green boxes represent single negotiation text process. Blue boxes represent components used from the Delphi method. Black boxes represent post-agreement processes.

Experts were selected by the CBC based on their individual and collective expertise regarding: biosafety measures, epidemiology, public health, and ID prevention. In addition, experts were selected based on their experience with laboratory- or field-based research, or both. Experts were at liberty to select BSL measures as they deemed them appropriate to prevent coronavirus transmission in home and street environments. In addition, experts were at liberty to select COVID-19 prevention measures that have been published elsewhere. The search methods used by the authors to find relevant BSL and COVID-19 prevention measures were not suggested or enforced by the process, and, therefore, there may have been variations between experts in that respect. Experts had total independence to adapt their chosen BSL measures from the laboratory to the home/street environment. The CBC coordinated the creation of the first draft of the document that served as the basis for the Delphi-styled SNT process that ensued (Figure 1).

A total of 12 experts were invited to review the protocol. The research and professional expertise of the selected experts, as well as their role in the development of the biosafety-at-home flyer, is shown in Table 1. Experts had a combined 200+ years of experience in epidemiology, virology, ID prevention, and public health. At least four members of the expert committee are currently involved in COVID-19–related responses ranging from clinical and laboratory diagnoses to epidemiological and public health activities. At least four of our selected experts have worked in U.S. military scientific facilities.

**Table 1:** Role, professional, and research expertise of selected experts

Expert	Role	Field of experience				Years of experience		
		Biosafety measures	Epidemiology	Public health	Infectious disease prevention	Laboratory	Field	Clinical
Expert 1	A	X	X	X	X	> 40	> 40	–
Expert 2	A	X	X	X	X	28	28	–
Expert 3	A	–	–	X	–	–	> 25	–
Expert 4	A	X	X	X	X	3	12	3
Expert 5	A	X	X	X	–	5	5	–
Expert 6	A	X	–	–	X	–	–	25
Expert 7	R	–	X	X	X	0	9	–
Expert 8	R	X	–	X	–	22	–	–
Expert 9	A	X	–	X	X	14	14	–
Expert 10	A	X	X	X	X	15	9	24
Expert 11	A	X	–	–	X	16	13	–
Expert 12	R	–	–	–	X	20	20	–

A = author (named); R = reviewer (anonymous). Note: The order of experts in this table does not reflect the order of authors on this article nor the order of authors in the biosafety-at-home flyer shown in the Supplementary Materials. Expertise was self-reported or inferred from CVs and publicly available expertise information.

Experts devised many ingenious forms to adapt laboratory and research protocols to the home/street environment. For instance, experts proposed creating three areas (black, gray, and white) in the house to mimic decontamination areas in the research laboratory (see Supplemental Materials, Flyer S1). Experts provided suggestions on how to move from one area to the next, having in mind the contextual reality of home living. Researchers also provided advice on precautions to have in mind while venturing outside home, taking into consideration that most people will likely use public transportation (see Supplemental Materials, Flyer S1). Finally, researchers constantly offered well-known COVID-19 social distancing prevention measures: keep 2 m apart from other people, avoid crowded areas and large gatherings, stay at home (unless absolutely necessary), use face masks in public, and clean and disinfect frequently touched surfaces.^15^

The protocol document underwent 11 iterations. The final document was revised and approved by all coauthors and the two anonymous contributors. The 10th version was sent to a graphic designer to produce a user-friendly flyer (Sofía Baus, BausBox, Quito, Ecuador), according to the model shown in Figure 1. This eleventh revision, the biosafety-at-home flyer, was ready for distribution on May 2, 2020. Since then, the flyer has been distributed via personal or institutional social media platforms linked to the coauthors, other contributors, and digital newspapers and social media interest groups (Table 2). Some of the social media platforms used included Facebook, WhatsApp, and Instagram. Institutionally, the protocol was distributed via official websites or mass communication institutional emails. From these initial distributions, the flyer has been redistributed at a rate that cannot be fully measured, in part because of the fact that some re-share/impact is not easily accessible (such as WhatsApp forwarding). Nonetheless, from what is publicly available, we estimate that ∼7,000 people were reached in the first weeks of release (Table 2), not accounting for the redistributions that may have occurred from colleagues outside Ecuador. In addition, the CBC was invited to a radio interview to discuss COVID-19 prevention, including the biosafety-at-home flyer.

**Table 2:** Biosafety-at-home flyer distribution as of June 14, 2020

Distribution means	Reach
Social media—institutional profiles, number of shares	
Pontifical Catholic University of Ecuador	201
USFQ	79
CISeAL	61
Other*	212
Social media—personal profiles, number of shares	
All coauthors and their contacts	150
**Subtotal**	**703**
Institutional websites, readership	
CISeAL	∼3,000
Universidad San Francisco de Quito	∼3,000
**Subtotal**	**∼6,000**
**Total**	**∼6,700**

CISeAL = Center for Research on Health in Latin America; USFQ = Universidad San Francisco de Quito.

* Other includes a digital newspaper, biotechnology, and clinical laboratory interest social media groups.

In summary, we describe the use of a mixed-methods approach, based on principles from the SNT and DM, to develop consensus on how to translate research laboratory biosafety measures to the home/street environment. The resulting product, a user-friendly biosafety-at-home flyer, has been distributed via social media and emails. This biosafety protocol was developed for high-risk groups, including HCWs and people living in areas of intense SARS-CoV-2 transmission. Similar approaches can be used to adapt biosafety measures to local contextual realities in other areas of high SARS-CoV-2 transmission during this pandemic.

## Supplemental materials

Supplemental materials
