# Supplementary material for: Biosafety at Home: How to Translate Biomedical Laboratory Safety Precautions for Everyday Use in the Context of COVID-19
Source: Am J Trop Med Hyg. 2020 Jun 26;103(2):838–40. doi: 10.4269/ajtmh.20-0677 (PMC7410461; doi:10.4269/ajtmh.20-0677)
Supplement: Supplementary file 1 [file tpmd200677.SD1.pdf]

LEAVE/ENTER HOME SAFELY TO AVOID Severe Acute Respiratory Syndrome Coronavirus 2 (SARS-CoV-2)

A guide with recommendations for areas with intense SARS-CoV-2 community spread during restricted mobilization

Protocol aimed to medical personnel/staff, public with risk of exposure to COVID-19

- The severe acute respiratory syndrome coronavirus 2 (SARS-CoV-2) produces a severe respiratory disease called Coronavirus Disease 2019 **(COVID-19).**
- **SARS-CoV-2 is highly contagious through respiratory droplets and contact with contaminated surfaces.**
- The most widely accepted option to avoid infection is **proper hygiene including washing hands with water and soap frequently; decontaminating surfaces; and social distancing with an emphasis on people staying home.**
- Social distancing however is **unsustainable** over time. People need to go out to purchase food or medicines, complete bank transactions and work in essential functions.

These guidelines are based on **Standard Operating Procedures (SOPs)**, methodology used by governments of developed countries to provide technical procedures to staff, especially military personnel and health researchers, to regulate and systematize instructions, and ensure that procedures are conducted in the proper way to reduce errors.

Follow these instructions step by step carefully to avoid contaminating your home with SARS-CoV-2

AT HOME

1. Define **three adjacent areas** with non-moving imaginary boundaries. These areas could have physical barriers, such as walls or doors; or could be divided by invisible lines which must be respected. **Mark the boundaries with masking or color tape to make these lines visible.**

a. **White area.** - This is a 3-5 ft^2 clean area inside your house followed by the gray area.
b. **Gray area.** - This is a 3-5 ft^2 intermediate, decontamination area, inside your house.
(For preparing to leave the house OR to decontaminate yourself when returning to your house).
c. **Black area.** - This is the contaminated area located outside towards the main exit of the house that faces the street, which is assumed to be contaminated with the virus.

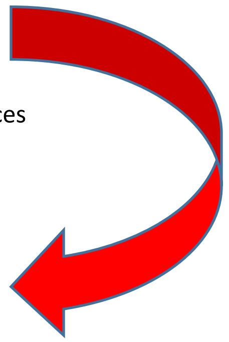

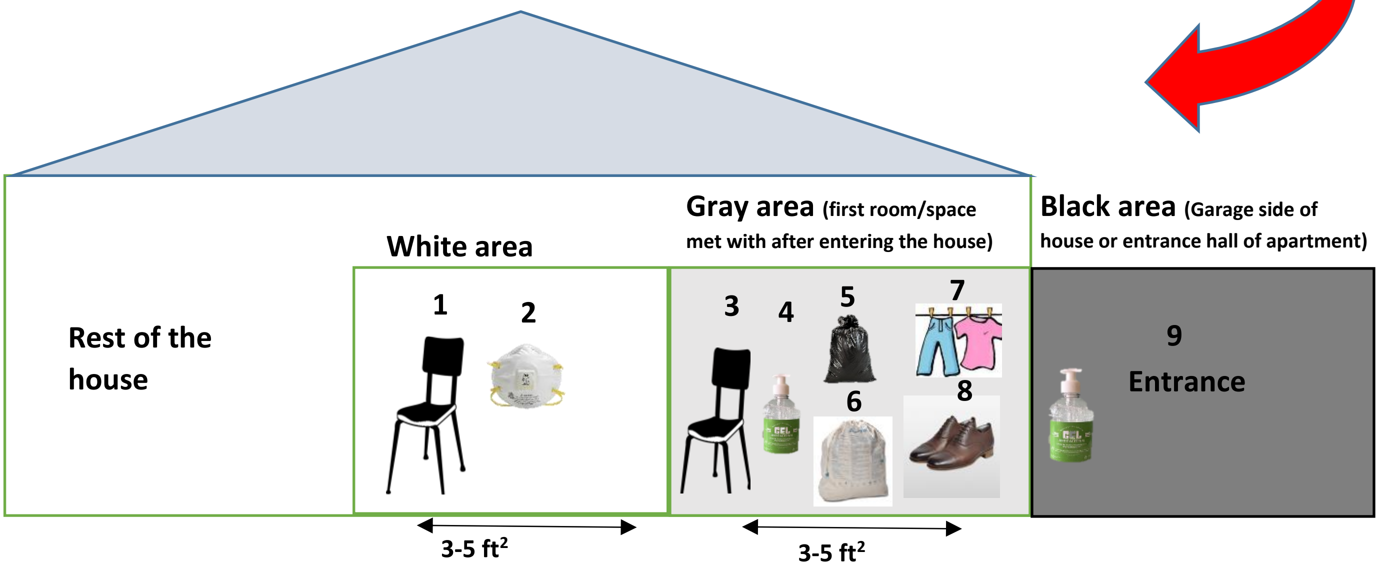

PLACE THE FOLLOWING ITEMS IN EACH AREA

WHITE AREA

- A chair ideally made of plastic or other easy-to-clean material **“1” (See figure above).** (Here you must place household clothing that you change out of when leaving the house or clothing you change into when entering the house. Clothes can either be placed down or hung).
- A protective facemask (**Ideally, an N95 mask, however, its use might be restricted in some countries or locations**) or cloth face covering.

GRAY AREA

- A chair ideally made of plastic or other easy to clean material **"3" (See figure above).**
- Hand sanitizer (with at least 60% alcohol content), antibacterial gel, rubbing alcohol (at least 70% Alcohol) or 70% Alcohol **"4". (See recipe in additional information below).**
- A plastic garbage bag **"5".**
- A clean bag or reusable shopping bag **"6".**
- Clothes to be used on the street (t-shirt/ sweater, shirt, pants, and socks) **“7”.** **“Street clothes” – A set of clothes that you will use while being outside the house. This set of clothes is to be changed into when leaving the house (see procedures to leave the house below) or change out of when entering the house (see procedures to enter the house below). Once you have returned from the outside and changed out of your set of “street clothes”, they should be washed (that means, you can use them for only one journey outside the house at a time – they need to be cleaned before using them again). It is recommended that you set apart at least 2-3 sets of “street clothes”.**
- Comfortable shoes that can be worn outside of the home **“8”.**

BLACK AREA

- Hand sanitizer (with at least 60% alcohol content), antibacterial gel or rubbing alcohol (at least 70% Alcohol) **"9" This can be left in the black area or can be taken with you to disinfect hands and exposed areas while on the street.**

PROCEDURE

ONLY RECOMMENDED IF INTENDED TO GO OUT TO PUBLIC AND ENCLOSED PLACES SUCH AS GROCERY STORES, SHOPS, AND BANKS. ALSO APPLY THIS PROCEDURE IF YOU NEED TO USE PUBLIC TRANSPORT WHEN LEAVING YOUR HOUSE.

IT IS NOT NECESSARY TO FOLLOW ALL OF THESE PROCEDURES IF GOING TO VERY OPEN SPACES WITH MORE THAN 6 FT. DISTANCE BETWEEN YOU AND ANY OTHER PERSON OR PET (I.E. PARKS OR WOODS).

TO LEAVE THE HOUSE

The procedure to leave the house is quite simple. The person leaving the house should perform the following actions below:

1. Enters the “WHITE AREA”.
2. Removes clothing worn in the house (shirt / t-shirt, pants, socks, and inside shoes) and places them on CHAIR (See Figure **“1” for a chair)** or hang them.
3. Dons the mask **“2”** on the face using appropriate technique and continue to the "GRAY AREA".
4. Dresses in street clothes (Item **"7"** in the figure) found on the CHAIR (item **"3"** in the figure) or hanging from a rack. Put on outside shoes (item **"8"** in the figure).
5. Exits the house, passing through the "BLACK AREA" into the street. When going out, there is nothing that you need to do in the “BLACK AREA”.

RECOMMENDATIONS on the use of facemask for asymptomatic people vary in each locality and country. Follow government or local authorities´ advice. HOWEVER, based on most recent evidence, we recommend that everyone going out the house should be wearing a facemask. In many shops or other facilities, its use is highly recommended or mandatory.

NOTE: If you are symptomatic or any person in your house is symptomatic, you SHOULD ONLY leave the house to seek medical care. In that case, a facemask SHOULD BE WORN to avoid spreading the virus.

CLOTHING RECOMMENDED FOR THE STREET

- Use shorts and a t-shirt/tank top under your street clothes.
- If you live in the highlands or any other cold areas, use a jacket with hood/hoodie that could be easily disinfected. If you leave in the coastland/Amazonian region or any other hot and humid area, use a light but full sleeve shirt and full-length pants.
- Use rubber boots or shoes that can be easily disinfected (AVOID SANDALS OR OPEN TOED FOOTWEAR).
- Rubber or disposable gloves are **NOT RECOMMENDED** EXCEPT if required to enter certain stores. The use of goggles may be mandatory too.

ON THE STREET

- Avoid touching the floor or surfaces that may be contaminated such as handrails, door knobs, banisters/railings, elevator buttons, door handles, metallic or plastic surfaces in buses or other public transportation, as much as possible, whenever it is safe to do so.
- Avoid shaking hands or direct contact with people (keep distance of at least 6 feet)
- Wash hands as often as possible/take alcohol gel or sanitizing gel with you and disinfect hands regularly.
- Avoid touching the floor/surfaces that could be contaminated (gravity forces virus particles to go down and could remain in surfaces for hours/days).
- Avoid touching your mask with your hands. Even though it is uncomfortable, touching the masks contaminates the face and the mask. Touching the mask also contaminates your hands if you are a carrier of the virus.

TO ENTER THE HOUSE

The person entering the house should perform the following actions below:

IN THE BLACK AREA

1. The person comes in from the street to the "BLACK AREA".
2. Open the house door and disinfect the doorknob with disinfectant **"9".**
3. Spray outside shoes´ sole with disinfectant or chrolorine bleach (see recipe below) **"9".**

IN THE GRAY AREA

4. Take one step into the "GRAY AREA.", remove the shoes **"8"** and leave them on the floor.
5. Remove street clothes (shirt / T-shirt, pants, and socks) **"7"** and place them in the plastic garbage bag **"5".** Ideally, do **NOT** re use the same set of street clothes before washing them. Alternatively, used clothes can be left in the "GRAY AREA" for at least 36 hours or be left in the sun for 12 hours.

.6 Remove mask using appropriate aseptic technique (i.e. release from the strings in the back while avoid touching the front of the facemask/face) and place it in the plastic garbage bag **"5".**

7. Proceed to disinfect with the antibacterial gel or 70% alcohol "**4;"** rub thoroughly hands, arms, exposed skin, and feet if you prefer.
8. Move to the ´´WHITE AREA´´.

IN THE WHITE AREA

9. Dress in your home clothing left behind when leaving. You can stay barefoot inside home or use new socks.
10. You are ready to enter the rest of your home safely.
11. Immediately wash hands with soap and water for at least 20 seconds. You can shower if you prefer, be sure to use lots of shampoo and body soap.

IF ENTERING THE HOUSE WITH BAGS OR FOR RECEIVING ORDERS

1. For grocery items, food or products bought or ordered that come in plastic bags; place/receive them in the ¨BLACK AREA¨.

2. Disinfect exterior of plastic bags by spraying disinfectant "9" like *Lysol* or chrolorine bleach or rubbing alcohol (70% alcohol).
3. Transfer the bags to the ¨GRAY AREA¨.
4. Remove canned or packaged items from plastic bags one at a time and spray or rub with 70% alcohol, hand sanitizer or any other disinfectant like *Lysol* or chrolorine bleach (see recipe below). Avoid disinfecting vegetables and fruits and open food.
5. As items are disinfected, place them in a clean plastic bag or reusable shopping bag and transfer them to the WHITE AREA. Items are now ready to enter the rest of your house.

Be sure to wash vegetables and fruits with abundant running water too before storing or consuming. Products that are going to be pealed for consumption, such as fruits, can be also be washed with soap.

ADDITIONAL INFORMATION

- If mask needs to be re used, **(not recommended)**, use gloves, remove it from plastic bag and place it in the oven for 30 minutes at 70ºC (CAUTION: N95 MASKS OR ANY OTHER MASK CONTAINING METAL SHOULD NOT BE DISINFECTED IN A MICROWAVE!!!). Homemade mask can be laundered or placed in soapy hot water for 10 minutes, then rinsed in hot water to remove soap. Air dry or dryer on hot 30 minutes or under the sun. You can also leave the mask in the ´´GRAY AREA´´ for 4 consecutive days without touching it or in the sun for 2 days. Only disinfected masks can again be placed in the ´´WHITE AREA´´.
- Put the used clothes in soapy water for at least 10 minutes before washing, preferably with hot water.
- Clean the floor and chair in the ´´WHITE AREA´´ and ´´GRAY AREA´´ regularly with alcohol or chrolorine bleach at least twice per week using the appropriate protection including gloves and if possible, a face mask.
- As disinfectant, use chrolorine bleach [add 2-3 tablespoons (10ml) of bleach (5-6%) to 1 liter of water]. AVOID MIXING BLEACH WITH ANYTHING EXCEPT WATER.
- 70 % ALCOHOL RECIPE: MIX 7.3 CUPS OF ETHYL ALCOHOL (95%) WITH 2.7 CUPS OF WATER IN A SPRAY DISPENSER.

ALWAYS REMEMBER TO

- MAINTAIN SOCIAL DISTANCING, AVOID CROWDS, TRY TO TRAVEL ALONE, ALWAYS KEEP A DISTANCE OF AT LEAST 6 FEET FROM OTHERS IN CLOSED ENVIRONMENTS AND 30 FEET IF PRACTICING AN EXERCISE LIKE RUNNING OR BIKING.
- MAINTAIN A DISTANCE FROM YOUR FAMILY AT HOME AS MUCH AS POSSIBLE; THIS WILL REDUCE THE RISK OF INFECTING THEM IF YOU ARE INFECTED.
- WASH HANDS OR USE HAND SANITIZER AS OFTEN AS POSSIBLE, EVEN AT HOME. THIS WILL REDUCE THE RISK OF ACQUIRING/SPREADING THE VIRUS
- IF DRIVING A VEHICLE DURING A PANDEMIC REMEMBER TO DISINFECT THE HIGH TOUCH SURFACES (DOOR HANDLES, STEERING WHEEL, GEAR SHIFTER AND ANY CONTROL YOU HAVE TOUCHED) AFTER RETURNING HOME AND BEFORE ENTERING THE HOME.

- SARS-CoV-2 MAIN FORMS OF TRANSMISSION ARE THROUGH DIRECT CONTACT WITH INFECTED PEOPLE THAT COULD BE ASYMPTOMATIC (THAT MAY LOOK HEALTHY BUT ARE INFECTED WITH THE VIRUS) OR CONTAMINATED OBJECTS.

This protocol was prepared and reviewed by: Renato Leon, Ph.D., Andres Carrazco, BS, Medical Entomology & Tropical Medicine Laboratory (LEMMT), Universidad San Francisco de Quito, (USFQ), Quito, Ecuador. William F. Waters, Ph.D. School of Public Health, USFQ, Quito, Ecuador. Michael J. Turell, Ph.D. Retired senior scientist, USAMRIID, Ft. Detrick, Frederick, MD, USA. Christian Fierro, MD. Metropolitan Hospital of Quito, Quito, Ecuador. Mario Grijalva, Ph.D. Tropical Disease Institute, Ohio University, Athens, OH., USA/Center for Research on Health in Latin America (CISeAL), Pontifical Catholic University of Ecuador, Quito, Ecuador. Derrick Mathias, Ph.D. Florida Medical Entomology Laboratory, University of Florida, Vero Beach, Fl., USA. Miguel Reina Ortiz, MD, Ph.D, CPH. College of Public Health, University of South Florida, Tampa, Fl., USA. Paul Suits, BS, MT, CIC., Stephen J. Thomas, MD, SUNY Upstate Medical University Infection Control, Syracuse, NY, EE. UU. Any queries or comments please send to rleon@usfq.edu.ec

Disclaimer: The biosafety procedures and guidelines contained herein were developed under the exclusive criteria of the authors. The statements do **NOT** necessarily represent the views or an official position of any institution or university.
