# Supplementary material for: Biosafety at Home: How to Translate Biomedical Laboratory Safety Precautions for Everyday Use in the Context of COVID-19
Source: Am J Trop Med Hyg. 2020 Jun 26;103(2):838–40. doi: 10.4269/ajtmh.20-0677 (PMC7410461; doi:10.4269/ajtmh.20-0677)
Supplement: Supplementary file 2 [file tpmd200677.SD2.pdf]

Universidad San Francisco de Quito

Pontificia Universidad Católica del Ecuador
CISeAL
CENTRO DE INVESTIGACIÓN PARA LA SALUD EN AMÉRICA LATINA
CENTER FOR RESEARCH ON HEALTH IN LATIN AMERICA

ENTRADA /SALIDA DEL HOGAR

MINIMIZANDO EL RIESGO DE CONTAGIO CON EL SARS-CoV-2, CAUSANTE DE COVID-19

GUÍA TÉCNICA Y RECOMENDACIONES PARA ÁREAS CON INTENSA TRANSMISIÓN COMUNITARIA DEL SARS-CoV-2 Y MOVILIZACIÓN RESTRINGIDA

Protocolo dirigido al personal MÉDICO/SANITARIO y público con riesgo de exposición a SARS-CoV-2

- El nuevo coronavirus responsable del síndrome respiratorio agudo severo **(SARS-CoV-2)** produce una enfermedad respiratoria grave llamada **(COVID-19).**
- **SARS-CoV-2 es altamente contagioso a través de micro gotas de saliva y contacto directo con superficies contaminadas.**
- Para evitar el contagio, se recomienda el **distanciamiento social** (quedarse en casa) y prácticas de limpieza e higiene como lavarse las manos frecuentemente y aplicar gel alcohol y desinfectantes en superficies potencialmente contaminadas.
- El aislamiento social **NO es sustentable en el tiempo.** La población tendrá que empezar a movilizarse para hacer compras de alimento, transacciones económicas o para su trabajo, aunque sea éste restringido.

Esta guía técnica-práctica fue preparada en base a los “Procedimientos de Operación Estándar” (POEs), metodología utilizada por los gobiernos de países desarrollados para proporcionar instructivos técnicos al personal e investigadores, especialmente de gobierno y militar, para sistematizar los procedimientos y asegurar que se realicen sin errores y de la manera más adecuada.

Siga estas instrucciones PASO A PASO para evitar contaminar su hogar con el coronavirus SARS-CoV-2 que produce COVID-19

EN SU HOGAR

1. Defina **tres áreas adjuntas** hacia la salida de su casa en donde pueda establecer límites fijos. Estas áreas pueden tener barreras físicas como puertas o paredes, también pueden dividirse con líneas imaginarias. **Marque las líneas con cinta adhesiva preferentemente de color para que sea visible.**

a. **Área blanca. -** Corresponde a un espacio de 1-1.5 m2 dentro de su casa en el pasillo de salida a continuación del cual se localizará el "ÁREA GRIS"

b. **Área gris. -** Corresponde a un espacio de 1-1.5 m2 al lado de la puerta de salida de su casa que será el área de **descontaminación**. (Aquí se preparará para salir a la calle o para entrar al hogar)

c. **Área negra. -** Corresponde al área potencialmente **contaminada** hacia afuera de su casa/ hacia el parqueadero o hacia fuera de la puerta de su casa.

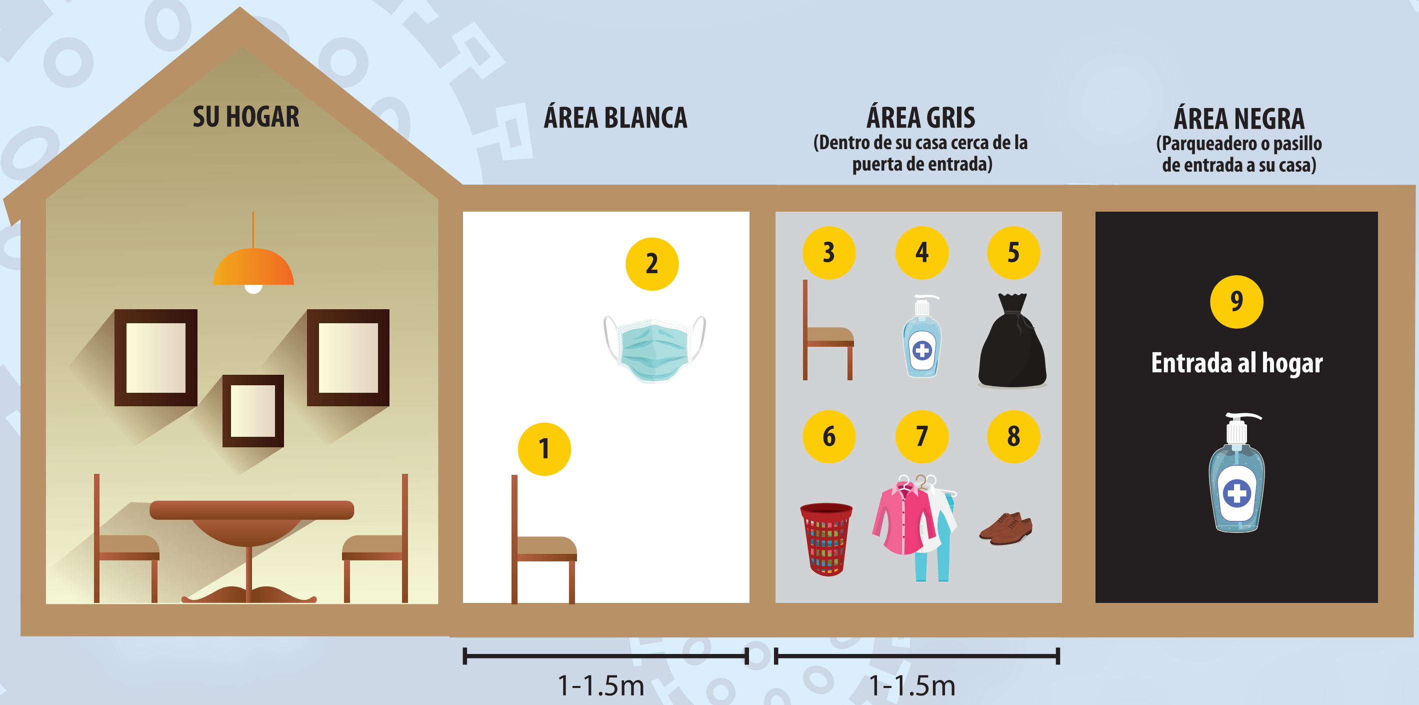

COLOQUE EN CADA ÁREA LO SIGUIENTE

ÁREA BLANCA

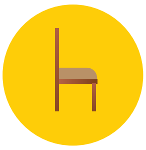
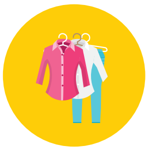
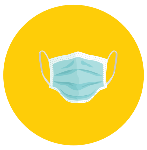

- Una silla preferiblemente hecha de plástico de fácil limpieza **“1” (Ver gráfico).**
- (En el “ÁREA BLANCA“ dejará su ropa de casa al salir y se volverá a colocar al entrar de la calle. Las prendas de casa pueden ser colocadas sobre la silla o colgadas en un armador.
- Una mascarilla sea N95 **(En algunos países o localidades PROHIBIDA para uso del público)** o quirúrgica, o de tela de fabricación casera.

ÁREA GRIS

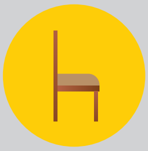
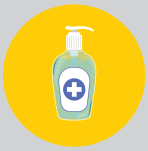
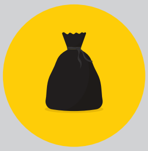
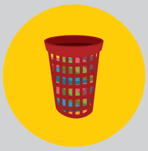
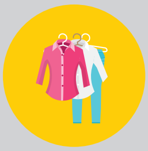
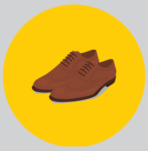

- Una silla preferiblemente hecha de plástico de fácil limpieza “3” (Ver gráfico).
- Gel alcohol (con al menos alcohol al 60%) o Alcohol industrial (Etanol) al 70% “4” Ver receta al último.
- Una bolsa plástica negra o de color “5”.
- Una cesta “6”.
- Ropa de calle (camiseta/saco de manga larga o chaqueta, pantalón y medias “7”. Evitar el uso de falda y ropa que exponga su piel.
- “Ropa de calle”: Un conjunto de ropa para usar en la calle. Colocarse estas prendas al salir de su casa (ver los procedimientos para salir de la casa que se describen abajo). Al llegar de la calle, dejar las prendas de calle en el “ÁREA GRIS“ (ver los procedimientos para entrar a la casa). La ropa de calle idealmente debe ser usada UNA SOLA VEZ y lavada después de regresar de la calle. También puede dejar reposar al menos 36 horas sin usarse o al sol por 12 horas. Se recomienda separar al menos 2-3 juegos de “ropa de calle”.
- Zapatos cómodos que puedan ser usados fuera de casa “8”.

ÁREA NEGRA

Gel alcohol (con al menos alcohol al 60%) o Alcohol industrial (Etanol) al 70% “9”. Éste puede permanecer en el “ÁREA NEGRA“ o si es posible llevarlo para desinfectar manos y piel expuesta o superficies que considere puedan contaminarse.

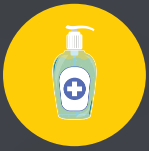

PROCEDIMIENTO

SE RECOMIENDA SOLO EN CASO DE DIRIGIRSE A ZONAS CON AGLOMERACIÓN DE PERSONAS, ÁREAS PÚBLICAS, AMBIENTES CERRADOS CON POCA VENTILACIÓN, TIENDAS, SUPERMERCADOS Y BANCOS. TAMBIÉN SE APLICA SI REQUIERE UTILIZAR TRANSPORTE PÚBLICO.

NO SEGUIR ESTE PROCEDIMEINTO SI SE DIRIGE A ZONAS ABIERTAS EN DONDE PUEDA MANTENER AL MENOS UNA DISTANCIA DE 2 METROS CON OTRAS PERSONAS (PARQUES, JARDINES Y BOSQUES).

PARA SALIR DE LA CASA

El procedimiento es muy sencillo. La persona que sale de la casa debe hacer lo siguiente:

1. Entrar al “ÁREA BLANCA“.

2. Quitarse la “ropa de casa“ (Camiseta/camisa, pantalón, medias y zapatos de casa) y colocar ropa en la silla o armador y zapatos en el suelo (Ver Figura **“1” la silla).**

3. Colocarse la mascarilla “**2”** usando una técnica apropiada. Continuar al “ÁREA GRIS“.

4. Vestirse con la “ropa de calle“ **“7” (Ver gráfico).** que se puede encontrar en la silla “3” o colgada **(Ver gráfico)**. Colocarse los zapatos **“8”.**

5. Salir de la casa por el “ÁREA NEGRA“ hacia la calle. NO NECESITA HACER NADA MÁS PARA SALIR.

ROPA QUE SE RECOMIENDA PARA LA CALLE

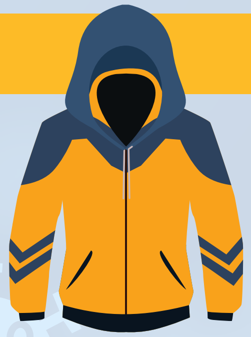

Usar en lo posible shorts y camiseta interior/playera/bividí debajo de la ropa de calle.

En zonas de altitud y clima frío, use una chaqueta preferiblemente impermeable con capucha que pueda ser desinfectada fácilmente. Si vive en zonas cálidas, use camiseta o camisa de manga larga y pantalón largo.

Use en lo posible botas de caucho o calzado que pueda ser desinfectado fácilmente (EVITE LAS SANDALIAS O CALZADO QUE EXPONGA LOS DEDOS DE LOS PIES).

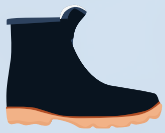

EL uso de guantes **NO SE RECOMIENDA EXCEPTO** cuando sea obligatorio, por ejemplo, en algunos supermercados. El uso de gafas de seguridad puede ser obligatorio también.

EN LA CALLE

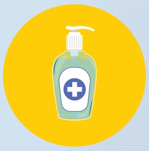

Evite tocar el suelo y superficies que puedan estar contaminadas como pasamanos, perillas de puertas, botones del ascensor, manijas de puertas, superficies metálicas o plásticas sea en buses o taxis. Si no puede evitarlo, trate de lavarse las manos o usar gel alcohol para desinfectarse a la brevedad posible.

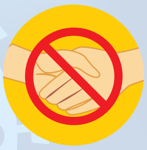

Evite saludar con la mano o con un abrazo o beso. Mantenga una distancia de **al menos 2 metros** con otras personas siempre.

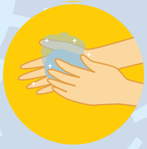

Lávese las manos frecuentemente usando abundante agua y jabón o desinfecte sus manos con gel alcohol regularmente.

Evite tocar superficies que puedan estar contaminadas (Las partículas virales caen por gravedad hacia abajo y contaminan superficies por horas o días).

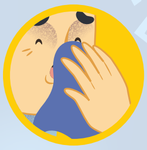

Evite tocar su mascarilla, ni sacarse/ponerse la misma varias veces. A pesar de la incomodidad y que dificulta su respiración, evitar manipular la superficie de la misma; esta puede estar contaminada y se podría contaminar sus manos o cara. Si sus manos se contaminan y manipula la mascarilla, éstas pueden también contaminarla.

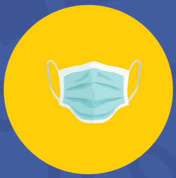

RECOMENDACIONES en el uso de la mascarilla para personas sin síntomas de COVID-19 varían de acuerdo al país y localidad. Por favor seguir las instrucciones de autoridades locales, SIN EMBARGO, de acuerdo a últimas evidencias, se recomienda que se utilice, SIEMPRE, mascarilla para salir a la calle. En algunos locales como supermercados el uso de mascarilla puede ser obligatorio.

NOTA: Si presenta síntomas o es COVID-19 positivo, NO DEBE SALIR DE SU CASA EXCEPTO SI NECESITA ATENCIÓN MÉDICA URGENTE. En este caso, el uso de mascarilla es OBLIGATORIO para evitar dispersar el virus en su comunidad.

PARA ENTRAR A LA CASA

Para ingresar a su casa debe seguir paso a paso las siguientes instrucciones:

EN EL ÁREA NEGRA

1. Llegar de la calle al "ÁREA NEGRA".
2. Usar paño húmedo para girar la perilla y abrir la puerta de calle o desinfectarse manos después de abrir la puerta.
3. Rociar alcohol "9" y desinfectar la suela de los zapatos.

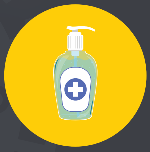

EN EL ÁREA GRIS

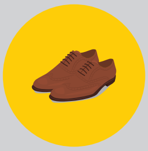

4. Dar un paso hacia el "ÁREA GRIS". Sacarse los zapatos "8" y colocarlos en el suelo.

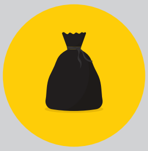

5. Sacarse la ropa de calle"7" (camiseta / camisa, pantalón y medias) y colocar en la funda plástica negra o de color "5". Idealmente, NO re-utilizar la misma ropa sino lavar o dejar reposar en el "ÁREA GRIS" por al menos 36 horas o dejar en el sol por 12 horas.

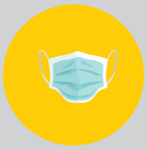

6. Remover la mascarilla de su cara utilizando una técnica aséptica adecuada (liberarla jalando de las tiras elásticas o desanudando las tiras de tela SIN TOCAR el frente de la mascarilla) y colocar en la funda plástica negra o de color "5".

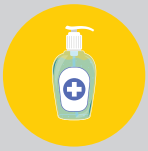

7. Desinfectar con gel alcohol o alcohol al 70% manos, brazos, piel expuesta, y los pies si desea.

8. Pasar al "ÁREA BLANCA".

EN EL ÁREA BLANCA

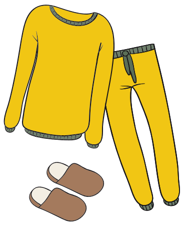

9. Vístase con su ropa de casa que dejo al salir. Puede permanecer descalzo o usar medias de casa.

10. Está listo para seguir hacia el resto de su hogar.

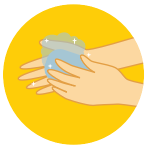

11. Lávese inmediatamente las manos por al menos 20 segundos o tome una ducha si prefiere. Utilice abundante jabón y shampoo.

PARA INGRESAR CON COMPRAS O PEDIDOS A DOMICILIO

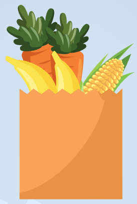

1. Llegar con las compras o recibir sus pedidos en el "ÁREA NEGRA". Si los productos vienen en fundas plásticas, rociar el exterior rápidamente con alcohol al 70% o un desinfectante **"9"** como por ejemplo *Lysol.*

2. Transferir fundas con los productos al "ÁREA GRIS".

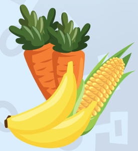

3. Remover productos de las fundas y de cualquier envoltura y desinfectar uno por uno en lo posible una vez más usando desinfectante. Evite desinfectar directamente vegetales, frutas o comida descubierta.

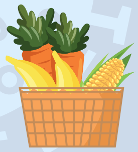

4. Transferir los productos en una canasta o cesta limpia al "ÁREA BLANCA". Los productos están listos para ingresar al hogar.

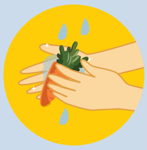

Asegurarse de lavar los vegetales y frutas con abundante agua; y con agua y jabón los productos que se puedan pelar como las frutas.

INFORMACIÓN ADICIONAL

La mascarilla de tela o de fabricación casera puede ser lavada en agua caliente con detergente por 10 minutos, luego enjuagarse en agua caliente también para eliminar el jabón. Puede secar con un secador con aire caliente por 30 minutos o también bajo el sol. También se puede dejar la mascarilla en el "ÁREA GRIS" por 4 días consecutivos sin tocarla o al sol por 2 días. Solo las mascarillas desinfectadas pueden volver a colocarse en el "ÁREA BLANCA".

- Coloque la ropa de calle usada en agua con detergente en una tina por al menos 10 minutos antes de lavar, preferentemente en agua caliente.
- Limpie el piso y la silla en el "ÁREA BLANCA" y "ÁREA GRIS" regularmente con alcohol o cloro al menos dos veces por semana usando la protección adecuada, incluidos guantes y, si es posible, una mascarilla.
- Como desinfectante, agregue 2-3 cucharadas de cloro (10 ml) al 5-6% a 1 litro de agua.
- EVITE MEZCLAR EL CLORO CON CUALQUIER COSA EXCEPTO EL AGUA.
- RECETA DEL ALCOHOL AL 70%: MEZCLAR 7 TAZAS DE ALCOHOL INDUSTRIAL Y 3 TAZAS DE AGUA EN UN RECIPIENTE PREFERIBLEMENTE EN UN DISPENSADOR EN SPRAY (ROCIADOR).

NO OLVIDAR

MANTENER DISTANCIA SOCIAL, EVITAR LAS MULTITUDES, TRATAR DE VIAJAR SOLO, SIEMPRE MANTENER UNA DISTANCIA DE AL MENOS 2 METROS DE OTRA PERSONAS EN ENTORNOS CERRADOS Y AL MENOS 6 METROS SI PRACTICA UN EJERCICIO COMO CORRER O BICICLETA.

MANTENER LA DISTANCIA CON SU FAMILIA EN SU HOGAR, ESTO REDUCIRÁ EL RIESGO DE TRANSMITIR EL COVID-19 SI USTED ESTA INFECTADO.

LAVARSE LAS MANOS O UTILIZAR GEL ALCOHOL FRECUENTEMENTE, INCLUSO EN SU CASA; ESTO REDUCIRÁ EL RIESGO DE ADQUIRIR O PROPAGAR EL VIRUS.

SI CONDUCE UN VEHÍCULO DURANTE LA PANDEMIA, DESPUÉS DE REGRESAR DE LA CALLE Y ANTES DE ENTRAR A LA CASA, DESINFECTAR LAS SUPERFICIES DE ALTO RIESGO (MANILLAS DE PUERTA, VOLANTE, PALANCA DE CAMBIOS, Y CUALQUIER BOTÓN QUE HAYA TOCADO).

LAS PRINCIPALES FORMAS DE TRANSMISIÓN DEL SARS-CoV-2, QUE PRODUCE COVID-19, SON A TRAVÉS DEL CONTACTO DIRECTO CON PERSONAS INFECTADAS, QUE PODRÍAN SER ASINTOMÁTICAS (QUE PUEDEN PARECER SALUDABLES, PERO ESTÁN INFECTADAS CON EL VIRUS) O MEDIANTE CONTACTO DIRECTO CON OBJETOS CONTAMINADOS.

Este protocolo fue preparado y revisado por: Renato León, PhD., Andrés Carrazco, Ing. Biotecnología, Laboratorio de Entomología Médica & Medicina Tropical (LEMMT), Universidad San Francisco de Quito, (USFQ), Quito, Ecuador. William F. Waters, PhD. Escuela de Salud Pública, USFQ, Quito, Ecuador. Michael J. Turell, PhD. Investigador retirado, USAMRIID, Ft. Detrick, Frederick, MD, EE. UU. Christian Fierro, MD. Hospital Metropolitano de Quito, Quito, Ecuador. Mario Grijalva, PhD. Instituto de Enfermedades Tropicales, Universidad de Ohio, Athens, OH., EE. UU. / Centro de Investigación para la Salud en América Latina (CISeAL), Pontificia Universidad Católica del Ecuador, Quito, Ecuador. Derrick Mathias, PhD. Laboratorio de Entomología Médica de Florida, FMEL, Universidad de Florida, Vero Beach, Florida, EE. UU. Miguel Reina Ortiz, MD, PhD, CPH. Colegio de Salud Pública, Universidad del Sur de la Florida, Tampa, Florida, EE. UU. Paul Suits, BS, MT, CIC., Stephen J. Thomas, MD, SUNY Upstate Medical University Infection Control, Syracuse, NY, EE. UU.

Cualquier consulta o comentario favor enviar a rleon@usfq.edu.ec

Descargo de responsabilidad: El protocolo y procedimientos de bioseguridad presentados en este documento son de responsabilidad de los autores. NO representan necesariamente a una posición de ninguna institución o universidad.

Diseño y Diagramación
una colaboración
de Sofía Baus

09 9706 4204
bausbox@gmail.com
@bausbox
Web: bausbox.wixsite.com/sofiabaus

BAUS BOX espacio creativo
